# 936. Risk Factors and Clinical Outcomes for Extended-Spectrum Beta-Lactamase Producing Enterobacterales Blood Stream Infections in Patients with Hematologic Malignancies

**DOI:** 10.1093/ofid/ofab466.1131

**Published:** 2021-12-04

**Authors:** Amanda Lefemine, Jacqueline Meredith, Katie Hammer, Ekaterina Kachur, Jiaxian He, Carly C Speight, zainab shahid

**Affiliations:** 1 Atrium Health’s Carolinas Medical Center, Charlotte, North Carolina; 2 Atrium Health, Carolinas Medical Center, Charlotte, North Carolina; 3 Atrium Health, South Carolina; 4 Genentech, Fort Mill, South Carolina; 5 Atrium Health-Levine Cancer Institute, Charlotte, North Carolina; 6 University of North Carolina, Eshelman School of Pharmacy, Charlotte, North Carolina; 7 Levine Cancer Institute, Charlotte, North Carolina

## Abstract

**Background:**

Hematologic malignancy patients have high rates of antibiotic exposure, and increasing resistance is a major concern, particularly with extended-spectrum beta lactamases (ESBL) in Enterobacterales blood stream infections (BSIs). Identifying risk factors for ESBL-producing Enterobacterales (ESBL-E) BSIs may facilitate faster appropriate antibiotic use and decrease mortality.

**Methods:**

This was a retrospective study of patients with hematologic malignancies and *Escherichia coli* or *Klebsiella spp.* bacteremia admitted to Carolinas Medical Center in Charlotte, NC from January 2010 through September 2020. The primary objective was to compare 30-day mortality rates for patients with ESBL-E BSIs to those with non-ESBL-E BSIs. Fisher’s exact or Mann-Whitney U tests were used for primary and secondary clinical outcomes as appropriate. Risk factors associated with 30-day mortality and ESBL production were assessed as secondary objectives using logistic regression models.

**Results:**

A total of 28 patients with ESBL-E BSIs and 60 patients with non-ESBL-E BSIs were included. The 30-day mortality rate with ESBL-E BSIs was 25% compared to 15% with non-ESBL-E BSIs (P = .373). In-hospital mortality, 30-day infection recurrence, intensive care unit (ICU) admission, and length of stay after culture were not significantly different. However, time to optimal therapy was longer in the ESBL-E group (median 42.3 vs 1.9 hr; P < .001). Multivariate logistic regression analysis showed an association of 30-day mortality with ICU admission (OR 16.7; 95% CI, 3.56-78.4; P < .001) and longer time to optimal therapy (OR 1.03; 95% CI, 1.0-1.05; P = .026). Prior ESBL-positive culture was associated with ESBL-E BSI in the univariate logistic regression (OR 9.83; 95% CI, 1.05-92.56; P = .046). Additionally, prolonged neutropenia (OR 3.05; 95% CI, 1.01-9.23; P = .049) and prior intravenous antibiotic use (OR 2.96; 95% CI, 0.96-9.09; P = .059) were associated with ESBL-E BSI in the multivariate analysis.

**Conclusion:**

Significantly longer time to optimal therapy was seen in ESBL-E BSIs and was associated with mortality in patients with hematologic malignancies. The identified ESBL risk factors create an opportunity to decrease delay in optimal therapy through risk stratification during initial antibiotic selection.

**Disclosures:**

**Ekaterina Kachur, PharmD, BCOP**, **Bristol Myers Squibb** (Advisor or Review Panel member)**Genentech** (Employee)**Glaxosmithkline** (Advisor or Review Panel member)**Kyowa Kirin** (Advisor or Review Panel member)

